# RBPMS::NTRK3-rearranged sacral malignant spindle cell tumor in neurofibromatosis diagnosed by RNA sequencing: a case report and literature review

**DOI:** 10.3389/fmed.2026.1914327

**Published:** 2026-08-27

**Authors:** Chaopeng Chen, Bin Qi, Yuanyuan Miao, Zhujiang Zhuang, Chaowen Wang, Weiping Dai

**Affiliations:** Department of Pathology, Central Hospital of Guangdong Provincial Nongken, Zhanjiang, Guangdong, China

**Keywords:** entrectinib, malignant peripheral nerve sheath tumor, neurofibromatosis, NTRK3, NTRK-rearranged spindle cell neoplasm, pan-TRK, RBPMS::NTRK3 fusion, RNA sequencing

## Abstract

Malignant spindle cell tumors arising in patients with neurofibromatosis are diagnostically challenging because malignant peripheral nerve sheath tumor (MPNST) and emerging NTRK-rearranged mesenchymal neoplasms may show overlapping clinical, histological, and immunophenotypic features. We report a 31-year-old man with a more than 20-year history of neurofibromatosis who presented with progressive lumbosacral pain and left lower-limb numbness. Imaging revealed a large cystic-solid sacral mass involving the S1–S2 region and extending through the sacral foramina into the pelvis. Core needle biopsy showed an intermediate-grade malignant spindle cell tumor with partial CD34 expression, strong pan-TRK positivity, increased p53 expression, and a Ki-67 labeling index up to 50% in hotspot areas. NTRK1 break-apart fluorescence *in situ* hybridization (FISH) was negative; however, RNA-based next-generation sequencing identified an RBPMS::NTRK3 fusion, establishing a molecularly confirmed NTRK3-rearranged spindle cell neoplasm and explaining the pan-TRK positivity as a true-positive result. The negative NTRK1 FISH reflected involvement of NTRK3 rather than NTRK1, illustrating that single-gene FISH cannot exclude an NTRK fusion. Given the large local tumor burden, neurological symptoms, high surgical morbidity, and the confirmed actionable fusion, the patient received molecularly guided entrectinib combined with palliative radiotherapy, with marked pain relief, neurological improvement, and radiological tumor shrinkage; at the most recent follow-up, approximately 7 months after starting entrectinib, he remained without evidence of disease progression. This case underscores that pan-TRK positivity should prompt confirmatory RNA-based sequencing rather than reliance on single-gene FISH; that actionable NTRK fusions may occur in the neurofibromatosis setting, where MPNST is the principal differential; and that molecularly informed multimodal therapy can benefit anatomically complex tumors, although response attribution requires caution when targeted therapy and radiotherapy are combined.

## 1 Introduction

Neurofibromatosis is a tumor-predisposition syndrome characterized by multiple neural, cutaneous, and soft-tissue lesions. Among its clinically important complications, MPNST is a major concern because of its aggressive behavior and close association with neurofibromatosis type 1 (NF1). MPNST accounts for approximately 5%–10% of sarcomas, and the lifetime risk of MPNST in patients with NF1 has been estimated at approximately 8%–13% (1–3). Therefore, the development of a painful, enlarging, or deep-seated mass in a patient with neurofibromatosis should prompt careful evaluation for malignant transformation.

Malignant spindle cell tumors arising in the sacral and pelvic region are particularly difficult to classify. Their deep anatomical location, frequent extension through neural foramina or pelvic soft-tissue planes, and nonspecific neurological manifestations may obscure the diagnosis. Pathologically, “malignant spindle cell tumor” is a descriptive term rather than a specific entity, and the differential diagnosis includes MPNST, undifferentiated spindle cell sarcoma, synovial sarcoma, spindle cell carcinoma, spindle cell melanoma, and emerging molecularly defined mesenchymal tumors. Because MPNST itself shows considerable morphological and immunophenotypic heterogeneity, accurate classification often requires integration of clinical context, imaging, histopathology, immunohistochemistry, and molecular testing (4).

NTRK-rearranged spindle cell neoplasms have recently emerged as a molecularly defined group of mesenchymal tumors with therapeutic implications, recognized as an emerging entity in the 2020 World Health Organization classification of soft tissue and bone tumors (5). They span a broad histological spectrum, from lipofibromatosis-like neural tumors to higher-grade sarcoma-like lesions, and may overlap morphologically and immunophenotypically with MPNST and other spindle cell malignancies (5, 6). pan-TRK immunohistochemistry is a useful screening tool for tumors that may harbor NTRK fusions; however, pan-TRK positivity alone does not establish an NTRK fusion, and confirmatory molecular testing—preferably sequencing-based—is recommended, particularly when TRK inhibitor therapy is considered (7).

Here, we report a young man with long-standing neurofibromatosis who developed a large sacral malignant spindle cell tumor extending into the pelvis. Core biopsy showed an intermediate-grade malignant spindle cell tumor with strong pan-TRK positivity; NTRK1 break-apart FISH was negative, but RNA-based next-generation sequencing identified an RBPMS::NTRK3 fusion, establishing a molecularly confirmed NTRK3-rearranged neoplasm. The patient received molecularly guided entrectinib combined with palliative radiotherapy, with symptomatic improvement and radiological tumor shrinkage. Beyond documenting a rare fusion partner in the neurofibromatosis setting, this case illustrates a practical diagnostic lesson: a negative single-gene NTRK1 FISH result does not exclude an NTRK fusion, and RNA-based sequencing is essential for confirmation.

## 2 Case presentation

### 2.1 Patient information

A 31-year-old man was admitted to our hospital on October 10, 2025, because of a more than 20-year history of neurofibromatosis and progressive low back pain with numbness and pain in the left lower limb for more than 2 months. More than two decades earlier, he had developed multiple cutaneous and subcutaneous nodules without an identifiable trigger; the lesions gradually enlarged and became more widely distributed. He had undergone several surgical resections at outside hospitals, with histopathology confirming multiple neurofibromas.

In August 2025, the patient developed lumbosacral pain with numbness and pain radiating to the left lower limb. Magnetic resonance imaging (MRI) at an outside institution revealed multiple nodules and masses involving the pelvis, muscles, and vertebral bodies, consistent with neurofibromatosis. The largest lesion, on the left side of the sacrum, showed suspicious features for malignant transformation: a cystic-solid mass with heterogeneous enhancement extending through the sacral foramina and partially protruding into the pelvic cavity. On September 8, 2025, ultrasound-guided core needle biopsy of this lesion at the outside hospital suggested a low-grade malignant spindle cell tumor, and the patient was referred to our hospital for further evaluation and treatment.

At admission, he reported persistent lumbosacral pain and left lower-limb numbness and pain, partially controlled with oral oxycodone-containing analgesics (numeric rating scale score 3). He appeared mildly fatigued, with fair appetite and sleep, no obvious change in bowel or urinary function, and no marked recent weight loss. His Eastern Cooperative Oncology Group performance status was 1.

### 2.2 Physical examination

On admission, the patient was conscious and cooperative. Multiple cutaneous and subcutaneous nodules were observed over the trunk and limbs, consistent with neurofibromatosis; histopathology of previously resected nodules had confirmed neurofibromas. The diagnosis of neurofibromatosis had been established clinically more than 20 years earlier on the basis of progressively multiplying, histologically confirmed multiple neurofibromas; however, systematic documentation of additional cardinal diagnostic features (such as café-au-lait macules, skinfold freckling, or Lisch nodules) was not available in the referral records, and germline NF1 genetic testing was not performed. Mild tenderness was present over the lumbosacral region, with evident numbness and pain of the left lower limb. Muscle tone in both lower limbs was normal, and bilateral lower-limb muscle strength was approximately grade 5−. No definite bowel or bladder dysfunction was identified initially, and cardiopulmonary and abdominal examinations were unremarkable. During subsequent treatment, the patient developed mild swelling of the left lower limb and weakness of the left foot; after targeted therapy and radiotherapy the pain gradually improved, although residual left foot weakness persisted, and before discharge the left lower-limb muscle strength was approximately grade 4 with otherwise normal strength.

### 2.3 Laboratory and imaging findings

Initial laboratory evaluation included complete blood count, serum biochemistry, coagulation profile, infection screening, and tumor markers. The complete blood count showed an increased neutrophil ratio, reduced hematocrit, and decreased mean corpuscular volume, suggesting mild anemia. Serum biochemistry revealed slightly decreased sodium, urea, and creatinine. Urinalysis, stool examination, neurological tumor markers, pretransfusion infectious screening, interleukin-6, and coagulation tests showed no major abnormalities. Echocardiography demonstrated preserved left ventricular systolic and diastolic function without significant structural or Doppler abnormalities.

Computed tomography (CT) of the chest, abdomen, and pelvis revealed multiple solid pulmonary nodules; the larger lesion in the anterior basal segment of the right lower lobe was classified as Lung-RADS 4A, while the remaining lesions were considered more likely benign or inflammatory (Supplementary Figure S1A). Multiple cutaneous nodular shadows in the chest, abdomen, and gluteal region were consistent with neurofibromatosis, and multiple cystic-solid masses were detected in the sacrum, prompting further MRI (Supplementary Figure S1B). Lumbar and pelvic MRI showed extensive nodular and mass-like abnormal signals around the lumbar and sacral vertebrae (Supplementary Figure S1C) and multiple lesions in the skin and subcutaneous soft tissues of the lower abdomen, lower back, gluteal region, and upper thighs, consistent with neurofibromatosis (Supplementary Figure S1D). The largest lesion was at the S1–S2 level, involving the sacrum and sacral lamina and narrowing the sacral canal (Supplementary Figure S1E), requiring histological confirmation.

## 3 Diagnostic assessment

The outside biopsy had suggested a low-grade malignant spindle cell tumor. Immunohistochemistry showed CK negativity, partial CD34 positivity, retained INI-1 expression, ERG negativity, CD31 negativity, focal Actin/SMA positivity, Desmin negativity, TFE-3 negativity, S100 negativity, SOX10 negativity, partial loss of H3K27me3 expression, a Ki-67 index of approximately 20% in hotspot areas, and retained SMARCA4 and SMARCA2 expression, favoring a low-grade malignant spindle cell tumor with a recommendation for additional tissue.

At our hospital, repeat core needle biopsy of the left pelvic mass showed an intermediate-grade malignant spindle cell tumor (Supplementary Figures S2A, B). Immunohistochemistry demonstrated positivity for Vimentin, partial CD34 positivity, p16 negativity, approximately 70% p53 positivity, a Ki-67 index of approximately 50% in hotspot areas and 20% elsewhere, negativity for CAM5.2, CD68, and CD30, partial ALK positivity, focal SATB2 positivity, negativity for Myogenin, MyoD1, and BCOR, pan-TRK positivity, and CD99 positivity (Supplementary Figures S3A–H). The morphology and immunophenotype were considered suggestive of a spindle cell tumor with possible NTRK rearrangement, and soft-tissue fusion gene testing was recommended.

NTRK1 break-apart fluorescence *in situ* hybridization was negative, with no detectable NTRK1 rearrangement (Supplementary Figure S4). Because pan-TRK positivity together with a negative single-gene FISH result does not exclude a fusion involving NTRK2 or NTRK3, RNA-based next-generation sequencing was performed. Total RNA extracted from the formalin-fixed, paraffin-embedded tumor tissue (tumor cellularity 80%) was analyzed with a targeted RNA capture sequencing panel covering 105 fusion-related genes relevant to soft tissue and bone tumors (including NTRK1, NTRK2, and NTRK3). This panel was designed and validated exclusively for the detection of fusion transcripts; it does not provide quantitative gene expression profiling, and no matched adjacent normal tissue was sequenced for comparative expression analysis. Sequencing identified an RBPMS-exon5::NTRK3-exon14 fusion—a known oncogenic NTRK3 fusion classified as an actionable alteration—together with two fusions of unknown clinical significance (FUS-exon3::BCL11B-exon2 and BCOR-exon1::DMD-exon8). The RBPMS::NTRK3 fusion accounted for the strong pan-TRK immunopositivity, and the negative NTRK1 break-apart FISH was concordant, reflecting involvement of NTRK3 rather than NTRK1.

Integrating the morphology, immunophenotype, and molecular findings, the tumor was classified as a malignant spindle cell neoplasm harboring an RBPMS::NTRK3 fusion—a molecularly confirmed NTRK3-rearranged neoplasm—arising in the setting of neurofibromatosis. The differential diagnosis included MPNST, undifferentiated spindle cell sarcoma, spindle cell carcinoma, spindle cell melanoma, and synovial sarcoma. CK and CAM5.2 negativity argued against spindle cell carcinoma, and S100 and SOX10 negativity argued against a conventional Schwannian tumor; however, an NF1-associated MPNST harboring an NTRK fusion could not be entirely excluded, given the neurofibromatosis background and the partial loss of H3K27me3 expression. In either interpretation, the confirmed RBPMS::NTRK3 fusion established an actionable molecular basis supporting NTRK-directed therapy.

## 4 Clinical course and follow-up

The diagnostic and treatment timeline, including the most recent follow-up, is summarized in Table 1.

**Table 1:** Timeline of diagnosis, treatment, and follow-up.

Date	Phase/event	Clinical course, investigations, and findings
>20 yr before	History	Multiple cutaneous and subcutaneous neurofibromas with several prior resections at outside hospitals (histologically confirmed neurofibromas); long-standing neurofibromatosis.
Aug 2025	Symptom onset	Lumbosacral pain with left lower-limb numbness and pain. Outside MRI: multiple neurofibromatosis-related lesions; largest left sacral lesion suspicious for malignant transformation.
Sep 8, 2025	Outside biopsy	Ultrasound-guided core needle biopsy of the left sacral lesion: low-grade malignant spindle cell tumor. Referred to our hospital.
Oct 10, 2025	Admission	Persistent lumbosacral and left lower-limb pain (NRS 3); partial control with oral oxycodone; ECOG performance status 1.
Oct 13, 2025	Baseline imaging	CT: multiple solid pulmonary nodules (largest ∼11 mm × 10 mm, Lung-RADS 4A) and sacral cystic-solid masses (largest ∼104 mm × 91 mm). MRI: S1–S2 mass narrowing the sacral canal.
Oct 16, 2025	Repeat biopsy	Core needle biopsy (left pelvic mass): intermediate-grade malignant spindle cell tumor. IHC: strong pan-TRK+, partial CD34+, p53 ∼70%, Ki-67 ∼50%, S100−, SOX10−; partial H3K27me3 loss.
Oct 21, 2025	Procedure	Infusion port implanted.
Oct 28 – Nov 5, 2025	Molecular testing	NTRK1 break-apart FISH negative. RNA-based NGS (105-gene panel) identified an RBPMS-exon5::NTRK3-exon14 fusion (actionable), plus FUS::BCL11B and BCOR::DMD fusions of unknown significance — confirming an NTRK3-rearranged neoplasm.
Nov 9, 2025	Targeted therapy	Molecularly guided oral entrectinib 600 mg once daily initiated.
Nov 9–26, 2025	Entrectinib monotherapy window (∼17 days)	Symptomatic improvement in lumbosacral and left lower-limb pain before radiotherapy; no obvious drug-related adverse events.
Nov 26, 2025	Radiotherapy started	Palliative radiotherapy to the pelvic lesion: 40 Gy in 20 fractions, with a planned additional 20 Gy in 10 fractions.
Dec 8, 2025	Clinical response	Pain markedly decreased; sustained-release oxycodone no longer required; left lower-limb strength ∼grade 4−.
Dec 19, 2025	Imaging response	Pelvic MRI: tumor decreased to ∼91 mm × 76 mm (from ∼117 mm × 89 mm on prior MRI), with increased intratumoral necrosis.
Jan 5–16, 2026	Second radiotherapy phase	Reduced-field radiotherapy 20 Gy in 10 fractions; completed Jan 16 and discharged on continued entrectinib.
Feb 4, 2026	Follow-up (∼20 days post-RT)	CT: pulmonary nodules decreased (largest ∼8 mm × 5 mm); sacral masses smaller (largest ∼92 mm × 73 mm); several mildly enlarged pelvic lymph nodes noted (possible nodal involvement — to be monitored).
Jun 18, 2026	Latest follow-up (∼7 mo on entrectinib)	Routine follow-up: no evidence of disease progression; continuing oral entrectinib and remaining clinically stable.

CT, computed tomography; ECOG, Eastern Cooperative Oncology Group; FISH, fluorescence *in situ* hybridization; Gy, gray; IHC, immunohistochemistry; MRI, magnetic resonance imaging; NGS, next-generation sequencing; NRS, numeric rating scale; RT, radiotherapy. Entrectinib monotherapy preceded radiotherapy by ∼17 days, during which symptomatic improvement was already observed; an imaging response was first documented on December 19, 2025.

After admission, the patient received dehydration therapy to reduce tumor-related compression, together with analgesia, nutritional support, and other symptomatic management. On October 16, 2025, he underwent core needle biopsy of the left pelvic mass, and on October 21, 2025, an infusion port was implanted.

Given the large local tumor burden, sacral and sacral lamina involvement, neurological compression symptoms, high surgical morbidity, and the confirmed RBPMS::NTRK3 fusion, multidisciplinary discussion supported a combined approach using NTRK-targeted therapy and palliative radiotherapy. Oral entrectinib was initiated on November 9, 2025, at 600 mg once daily. Early during treatment, the patient reported improvement in lumbosacral and left lower-limb pain without obvious drug-related adverse events. On November 26, 2025, palliative radiotherapy to the pelvic lesion was started, with an initial plan of 40 Gy in 20 fractions to the gross tumor volume and an additional 20 Gy in 10 fractions depending on response and toxicity.

During combined treatment, the pain progressively improved. By December 8, 2025, lumbosacral and left lower-limb pain had decreased further, and the patient no longer required sustained-release oxycodone; examination showed left lower-limb muscle strength of approximately grade 4− and mild left-foot swelling. Pelvic MRI on December 19, 2025, showed that the sacral tumor had decreased in size, with a maximum cross-sectional dimension of approximately 91 mm × 76 mm, reduced from approximately 117 mm × 89 mm, and increased intratumoral necrosis. A subsequent MRI showed the lesion to be generally stable. On January 5, 2026, a second phase of reduced-field radiotherapy (20 Gy in 10 fractions) was begun, and by January 6, 2026, after nearly 2 months of entrectinib and 20 fractions of radiotherapy, the pain had largely resolved, muscle strength had improved, and MRI showed tumor shrinkage.

On January 16, 2026, the patient completed the second phase of radiotherapy and was discharged on continued oral entrectinib with regular follow-up; left gluteal and lower-limb numbness and pain had largely improved, although left-foot weakness persisted. On February 4, 2026, approximately 20 days after radiotherapy, follow-up CT showed that some pulmonary nodules had decreased in size (Supplementary Figure S5) and the cystic-solid sacral masses were slightly smaller, the largest measuring approximately 92 mm × 73 mm versus approximately 104 mm × 91 mm previously; several slightly enlarged pelvic lymph nodes were noted, warranting further assessment (Supplementary Figure S6). Overall, the patient showed symptomatic and radiological improvement after combined entrectinib and radiotherapy, with continued follow-up required to assess local control, possible nodal progression, and long-term durability.

At the most recent follow-up on June 18, 2026—approximately 7 months after the initiation of entrectinib—the patient remained on oral entrectinib with no evidence of disease progression and a stable clinical status.

## 5 Discussion

This case illustrates the diagnostic complexity of a malignant spindle cell tumor arising in a patient with long-standing neurofibromatosis. In this setting, MPNST is the principal differential diagnosis, being among the most clinically significant malignant complications of NF1 (1–3, 16). MPNST is itself morphologically and immunophenotypically heterogeneous, and its diagnosis requires clinicopathological correlation rather than reliance on any single feature (4, 17, 18). In our patient, the long-standing neurofibromatosis, deep sacral location, neurological symptoms, malignant spindle cell morphology, S100/SOX10 negativity, and partial loss of H3K27me3 were all compatible with MPNST, while partial CD34 expression and strong pan-TRK positivity raised the possibility of an NTRK-rearranged mesenchymal neoplasm, an emerging category in the 2020 WHO classification (5, 6, 19, 20).

Notably, co-expression of CD34 and S100 is an immunophenotypic pattern frequently observed in NTRK-rearranged mesenchymal neoplasms, having been reported in a substantial proportion of cases, particularly those showing a lipofibromatosis-like neural tumor morphology (6, 19, 20). Our case lacked this characteristic phenotype, demonstrating only partial CD34 positivity with complete S100 negativity. This discrepancy may be explained, at least in part, by differences in the fusion locus and partner gene: CD34/S100 co-expression has been described predominantly in NTRK1-rearranged tumors with partners such as LMNA and TPM3, whereas NTRK3-rearranged tumors, as well as higher-grade lesions across the NTRK-rearranged spectrum, tend to show a more variable immunophenotype in which S100 expression may be reduced or absent (8, 9, 19, 20). Given the rarity of RBPMS as a 5′ partner of NTRK3, the immunophenotypic correlates of this specific fusion remain undefined. Importantly, the absence of the CD34/S100 co-expression phenotype also implies that other differential diagnoses—most notably NF1-associated MPNST, which is typically S100-negative with H3K27me3 loss, as well as undifferentiated spindle cell sarcoma—cannot be readily excluded on immunophenotypic grounds alone, underscoring that the final classification in this case rests on the molecularly confirmed RBPMS::NTRK3 fusion rather than on immunohistochemistry.

The pivotal diagnostic step was molecular testing, and it carried an important methodological lesson. pan-TRK immunohistochemistry is a sensitive screen but is not specific for an NTRK fusion, and positive results require molecular confirmation (6, 7, 9, 22). In our patient, NTRK1 break-apart FISH was negative—a result that, taken alone, could have been read as excluding an NTRK fusion and might have denied the patient access to TRK-directed therapy. RNA-based next-generation sequencing, however, identified an RBPMS::NTRK3 fusion. The discordance is readily explained: a single-gene NTRK1 break-apart probe cannot detect rearrangements of NTRK2 or NTRK3, whereas RNA-based sequencing captures fusions across all three NTRK genes and diverse partners. This case thus provides a concrete demonstration that NTRK fusion testing is method-dependent and that a negative single-gene FISH result does not exclude an NTRK fusion; RNA-based sequencing should be the confirmatory modality when a TRK inhibitor is being considered (7, 21, 22). RBPMS is an uncommon NTRK3 fusion partner, and the RBPMS::NTRK3 fusion adds to the small but growing spectrum of NTRK3 partners reported in spindle cell and fibroblastic neoplasms.

The precise classification of this tumor lies at the boundary between two overlapping categories. Although the confirmed NTRK3 fusion supports an NTRK-rearranged spindle cell neoplasm, the neurofibromatosis background together with S100/SOX10 negativity, the absence of the characteristic CD34/S100 co-expression phenotype, and partial H3K27me3 loss leaves an NF1-associated MPNST harboring an NTRK fusion as a legitimate alternative. These categories are not mutually exclusive: Hiemcke-Jiwa et al. identified NTRK1 fusions (TPM3::NTRK1, LMNA::NTRK1, and CACYBP::NTRK1) in 3 of 21 NF1-related MPNSTs, indicating that actionable NTRK rearrangements can occur within tumors arising in an NF1-MPNST context (13). Conversely, pan-TRK positivity in MPNST may reflect neural differentiation rather than a true fusion and must be interpreted alongside molecular testing (14). Our case differs in that the NTRK fusion was molecularly confirmed; whether it is best regarded as an NTRK-rearranged spindle cell neoplasm or as an NF1-associated MPNST with an RBPMS::NTRK3 fusion, the actionable consequence—TRK-directed therapy—is the same.

Distinguishing definitively between these two possibilities would require knowledge of the germline NF1 status: confirmation of a pathogenic germline NF1 variant would place the tumor within a genetically predisposed setting, favoring an NF1-associated MPNST harboring a secondary NTRK3 fusion, whereas a negative result would support a sporadic NTRK-rearranged neoplasm arising coincidentally in a patient with clinically diagnosed neurofibromatosis. In our patient, germline DNA testing was not performed, as the molecular workup was confined to tumor-tissue RNA sequencing designed for fusion detection; the designation of neurofibromatosis therefore rests on the clinical phenotype and the histologically confirmed multiple neurofibromas, and germline NF1 testing would be a valuable addition to future follow-up.

The anatomical constraints of this tumor parallel previously reported deep pelvic and paraspinal NTRK-rearranged neoplasms. Yin et al. and Tsai et al. documented the morphologic diversity of NTRK-rearranged spindle cell neoplasms and emphasized that pan-TRK immunohistochemistry has limited specificity and requires molecular confirmation (8, 9). Cao et al. and Huang et al. described pelvic NTRK-rearranged spindle cell tumors with TPM3::NTRK1 fusions, the latter showing a marked response to larotrectinib that enabled subsequent surgery (10, 11). Zhou et al. reported a thoracic-spine NTRK-rearranged tumor with a SPECC1L::NTRK3 fusion that responded to entrectinib (12); notably, both that case and ours involve NTRK3, which single-gene NTRK1 FISH cannot detect. In our patient, the confirmed RBPMS::NTRK3 fusion provided a clear rationale for entrectinib, which is approved and guideline-recommended for NTRK fusion-positive solid tumors and has shown durable activity in molecularly confirmed disease (15, 23). Given the large sacral tumor burden and high surgical morbidity, molecularly guided entrectinib offered an organ-sparing alternative to extensive surgery, and the patient experienced marked pain relief, neurological improvement, and radiological tumor shrinkage.

It should be noted that the degree of tumor shrinkage in our patient was comparatively modest relative to the marked regressions frequently reported with TRK inhibitors, in which objective responses typically deepen over multiple treatment cycles (15, 23). Several factors may account for this deviation from the reported clinical experience. First, the initial response assessment was performed after only approximately 6 weeks of entrectinib, an interval likely too short to capture the best response, which in trial populations often develops over several months (15, 23). Second, the baseline tumor was very large (approximately 117 mm × 89 mm) with a cystic-solid architecture and osseous involvement of the sacrum and sacral lamina; bulky tumors with bony encasement have limited capacity for rapid volumetric collapse, and the increased intratumoral necrosis observed on follow-up MRI suggests a biological treatment effect that is underestimated by linear dimensional measurements alone. Third, the pivotal entrectinib trials were dominated by tumors harboring common fusions such as ETV6::NTRK3, whereas RBPMS is a rare 5′ partner whose dimerization capacity and downstream signaling output—and hence sensitivity to TRK inhibition—have not been specifically characterized. Fourth, the neurofibromatosis background raises the possibility of concomitant RAS-MAPK pathway activation through NF1 inactivation, which could theoretically provide bypass signaling and attenuate the response to TRK inhibition, although this was not molecularly evaluated in our patient. In addition, the concurrent palliative radiotherapy complicates attribution of the observed regression to TRK inhibition alone, as discussed below. Finally, the two co-occurring fusions of unknown significance (FUS::BCL11B and BCOR::DMD) cannot be excluded as biological modifiers of treatment response. Rather than undermining the rationale for TRK-directed therapy, these considerations highlight the uniqueness of this case—a rare fusion partner arising in an NF1 context within an anatomically constrained, bulky sacral tumor—and underscore the importance of longitudinal assessment beyond early dimensional change.

Several limitations should be acknowledged. First, entrectinib was administered concurrently with palliative radiotherapy, so the clinical and radiological improvement cannot be attributed to TRK inhibition alone; the most defensible interpretation is benefit from a combined, molecularly guided strategy. Second, the diagnosis rested on core biopsy rather than complete resection, which may limit assessment of intratumoral heterogeneity and grade. Third, although RNA-based sequencing confirmed the RBPMS::NTRK3 fusion, orthogonal DNA-based confirmation and functional validation were not performed, and the two co-occurring fusions of unknown significance (FUS::BCL11B and BCOR::DMD) were not further characterized. Moreover, the targeted panel used was designed solely for fusion detection and did not yield quantitative expression data, precluding assessment of tumor-specific gene expression enrichment relative to adjacent normal tissue; transcriptome-wide expression profiling would be required to address this question. Finally, although the patient has remained free of disease progression for approximately 7 months on entrectinib, longer follow-up is needed to confirm the durability of local control, the long-term neurological outcome, and the potential emergence of resistance.

## 6 Conclusion

This case documents a molecularly confirmed RBPMS::NTRK3-rearranged malignant spindle cell tumor arising in a patient with neurofibromatosis, in which pan-TRK positivity was a true-positive result and a negative single-gene NTRK1 break-apart FISH did not exclude the fusion. It underscores two practical messages: pan-TRK immunopositivity should prompt confirmatory RNA-based sequencing rather than single-gene FISH, because the fusion may involve NTRK2 or NTRK3; and actionable NTRK fusions may occur in the neurofibromatosis setting, where MPNST is the principal differential. Molecularly guided multimodal therapy provided meaningful clinical benefit in this anatomically complex tumor, although response attribution should remain cautious when targeted therapy and radiotherapy are combined. Integrating immunohistochemical screening with RNA-based sequencing enables accurate molecular classification and supports targeted treatment in this challenging scenario.

## Data Availability

The raw data supporting the conclusions of this article will be made available by the authors, without undue reservation.

## Supplementary material

SUPPLEMENTARY FIGURE S1 Imaging findings on admission. **(A)** Chest CT. **(B)** Multiple cystic-solid masses within the sacrum on CT. **(C)** Lumbar spine and pelvic MRI. **(D)** Multiple cutaneous and subcutaneous lesions in the lower abdomen, lower back, gluteal region, and upper thighs on MRI. **(E)** Sacral mass at the S1–S2 level with involvement of the sacrum and sacral lamina.

SUPPLEMENTARY FIGURE S2 Hematoxylin and eosin staining of the biopsy specimen (A, ×10; B, ×20). The tumor was composed of polygonal and oval cells with intermediate-grade features, arranged in intersecting fascicles with markedly increased cellularity, abundant eosinophilic cytoplasm, prominent nucleoli in some cells, and identifiable mitotic figures.

SUPPLEMENTARY FIGURE S3 Histopathological and immunohistochemical findings. Immunohistochemistry showed CD34 positivity **(A)**, p53 positivity in approximately 70% of tumor cells **(B)**, a Ki-67 labeling index of approximately 50% in hotspot areas and 20% elsewhere **(C)**, CD68 negativity **(D)**, ALK positivity in a subset of cells **(E)**, diffuse and strong pan-TRK positivity **(F)**, CD99 positivity **(G)**, and Vimentin positivity **(H)**.

SUPPLEMENTARY FIGURE S4 NTRK1 break-apart FISH showing no detectable rearrangement. FISH with an NTRK1 (1q22–q23.1) break-apart probe was performed on the sacral tumor; nuclei were counterstained with DAPI (blue). The assay was negative for NTRK1 break-apart, indicating no detectable NTRK1 rearrangement.

SUPPLEMENTARY FIGURE S5 Chest CT comparison between baseline and follow-up. The follow-up panel (February 4, 2026) shows multiple solid pulmonary nodules; the largest nodule had decreased compared with baseline (October 13, 2025), measuring approximately 8 mm × 5 mm versus approximately 11 mm × 10 mm.

SUPPLEMENTARY FIGURE S6 Follow-up CT changes of the sacral cystic-solid masses. Compared with baseline (October 13, 2025), follow-up CT (February 4, 2026) showed slight reduction of the cystic-solid sacral masses, the largest decreasing from approximately 104 mm × 91 mm to 92 mm × 73 mm; several enlarged pelvic lymph nodes were noted (largest approximately 12 mm × 8 mm).
